# Resveratrol-Induced Signal Transduction in Wound Healing

**DOI:** 10.3390/ijms222312614

**Published:** 2021-11-23

**Authors:** Anna-Lisa Pignet, Marlies Schellnegger, Andrzej Hecker, Michael Kohlhauser, Petra Kotzbeck, Lars-Peter Kamolz

**Affiliations:** 1 COREMED—Cooperative Centre for Regenerative Medicine, JOANNEUM RESEARCH Forschungsgesellschaft mbH, 8010 Graz, Austria; marlies.schellnegger@joanneum.at (M.S.); andrzej.hecker@joanneum.at (A.H.); michael.kohlhauser@stud.medunigraz.at (M.K.); petra.kotzbeck@joanneum.at (P.K.); lars-peter.kamolz@joanneum.at (L.-P.K.); 2 Research Unit for Tissue Regeneration, Repair and Reconstruction, Division of Plastic, Aesthetic and Reconstructive Surgery, Department of Surgery, Medical University of Graz, 8036 Graz, Austria

**Keywords:** resveratrol, wound healing, skin, molecular pathway, SIRT1 signaling

## Abstract

Resveratrol is a well-known polyphenol that harbors various health benefits. Besides its well-known anti-oxidative potential, resveratrol exerts anti-inflammatory, pro-angiogenic, and cell-protective effects. It seems to be a promising adjuvant for various medical indications, such as cancer, vascular, and neurodegenerative diseases. Additionally, resveratrol was shown to display beneficial effects on the human skin. The polyphenol is discussed to be a feasible treatment approach to accelerate wound healing and prevent the development of chronic wounds without the drawback of systemic side effects. Despite resveratrol’s increasing popularity, its molecular mechanisms of action are still poorly understood. To take full advantage of resveratrol’s therapeutic potential, a profound knowledge of its interactions with its targets is needed. Therefore, this review highlights the resveratrol-induced molecular pathways with particular focus on the most relevant variables in wound healing, namely inflammation, oxidative stress, autophagy, collagen proliferation and angiogenesis.

## 1. General Properties of Resveratrol

Resveratrol is a powerful nutritional polyphenol that has been found in more than 70 different plant species, such as grapes (skin and seeds) and various dietary sources, including red wine, peanuts, and soy [1]. Being produced by the innate host response system of plants, resveratrol protects them from fungal, bacterial and viral infections, as well as from harmful environmental influences such as UV radiation, ozone exposure and toxins [2]. However, as a nutraceutical resveratrol was shown to exhibit its beneficial properties also in human cells. In 1992 Renaud and De Logeril correlated the potential health benefits associated with moderate wine consumption- known as the so-called “French Paradox”- to wine polyphenols, specifically resveratrol [3]. Resveratrol has been receiving increasing scientific attention due to its extraordinarily broad spectrum of biological activities, including anti-oxidant, anti-inflammatory, and pro-angiogenetic properties [4,5,6]. Many of its effects may route in its anti-oxidative potential and also in the mimicry of caloric restriction (CR) [7,8]. Resveratrol was shown to promote longevity, similar to fasting. In yeast it extended the lifespan up to 70% by modulation of the Silencing Information Regulator 2 (Sirt2), the yeast ortholog of the mammalian Sirtuin 1 (SIRT1) [9]. It is also involved in cell proliferation, differentiation, and survival and possesses potent chemo-preventive and chemo-therapeutic properties. A body of studies has confirmed the inhibitory effects of resveratrol on carcinogenesis in all stages- initiation, promotion, and progression [10,11]. It displays effects towards a wide range of contemporary human diseases, such as cardiovascular diseases and neurodegenerative pathologies [12]. Resveratrol was also shown to exhibit beneficial properties on the human skin—it delays the aging process and reduces hypertrophic scar formation [7,13,14,15]. As aging and the formation of non-healing wounds are inseparably associated, there is increasing evidence that resveratrol may even promote wound healing [16].

## 2. Resveratrol in Wound Healing

The re-establishment of tissue integrity after injury is a complex and dynamic process consisting of four phases: inflammation, proliferation, maturation, and remodeling. The proliferation phase, which begins within days after injury, includes the major processes of healing, namely angiogenesis, collagen deposition, granulation tissue formation, and epithelialization [13]. As inflammation, oxidative stress and impaired wound bed perfusion are among the most relevant factors to delay wound healing, the antioxidant resveratrol seems to be a promising supportive treatment approach that interacts with the healing cascade on many levels [8,17,18]. Numerous studies provided hints that resveratrol might accelerate wound healing [8,19,20]. Tissue regeneration and revascularization in wounds are regulated by resveratrol-induced vascular endothelial growth factor (VEGF) expression and inhibited by the expression of pro-inflammatory factors such as tumor necrosis factor-α (TNF-α) [21,22,23]. Especially in microvasculopathy-associated ulcers such as diabetic foot ulcers, VEGF has a broad spectrum of wound healing-related activities, ranging from capillary growth to increased cell migration, collagen deposition, and epithelialization [22]. Pro-inflammatory markers such as Interleukin (IL)-1β, IL-6 and TNF-α prolong the inflammatory phase and delay healing [24,25]. An increase of these markers leads to an up-regulation of matrix metalloproteinases (MMP), which are especially elevated in chronic wounds [26]. MMPs excessively degrade local extracellular matrix (ECM) and thus impair cell migration in wounds [27]. In resveratrol-treated patients pro-inflammatory markers such as IL-1β, IL-6, TNF-α, MMP-2, -3, -9 and C-reactive protein (CRP) were shown to be reduced [28,29,30,31]. However, the exact resveratrol-mediated mechanisms of action leading to improved wound healing remain a mystery. The pleiotropic effects of resveratrol seem to be never ending with new potential targets discovered annually [32]. To fully benefit of its therapeutic potential, the understanding of its molecular interactions with its cellular targets is inevitable. This review highlights the most relevant pathways mediated or influenced by resveratrol that are related to cutaneous wound healing.

## 3. Resveratrol and SIRT1 Signaling

Several in vitro and in vivo studies reported resveratrol to act as SIRT1-upregulator, a NAD+ dependent histone deacetylase located in the cell nucleus. Although it is still not perfectly understood how resveratrol acts out its biological function, its activation of SIRT1 has been considered a key mechanism [33,34,35,36,37]. Seven sirtuin isoforms—SIRT1, SIRT2, SIRT3, SIRT4, SIRT5, SIRT6, and SIRT7—have been identified. All contain a conserved catalytic core domain composed of approximately 275 amino acid residues with variable N- and C-termini [38]. SIRT1 is the only sirtuin that is ubiquitously expressed [15]. It can be activated either via CR, or pharmacologically via CR-mimetics, such as nutritional or topically applied resveratrol [9]. Howitz et al. performed fluorescent deacetylation assays, which showed that resveratrol is able to lower the Michaelis constant (Km), and hence increase the binding affinity of SIRT1 for the acetylated substrate, as well as for NAD1+ [9]. The deacetylation rate of SIRT1 was doubled in the presence of 11 µM resveratrol and saturated at 100–200 µM of resveratrol. Hence, resveratrol displayed its effects in a dose dependent manner. Given the fact, that resveratrol seems to act on Km, but not on Vmax, it might be considered an allosteric effector, binding to the non-catalytic N-terminus of SIRT1 [9]. However, due to resveratrol’s poor bioavailability the question arises if its in vivo effects can be direct. In humans, the peak plasma levels of resveratrol and metabolites only reached about 2 μM after an oral dose of 25 mg [39]. Accordingly, Kaeberlein et al. stated that resveratrol-mediated effects on SIRT1 may rather be an artefact [40]. In their study they examined the activity of Sirt1 in the presence of resveratrol against their targeting peptides p53 and Histone 4. These peptides were either native or modified with Fluor de Lys. The deacetylation rate of Sirt1 in the presence of resveratrol was dependent on the fluorophore. No direct effects of resveratrol were observed. Additionally, they were not able to reproduce the longevity effect of resveratrol in different yeast strains. These findings indicate that resveratrol-mediated in vivo effects may not be based on direct SIRT1 activation but on a different molecular mechanism [41]. Pacholec et al. aimed to test, whether the attached fluorophore mimics a hydrophobic pocket on the native full-length protein substrates that provokes a higher affinity for SIRT1 [40]. The authors used a native peptide substrate and performed direct detection and quantification methods, such as high performance liquid chromatography (HPLC). Their results showed that resveratrol does not activate SIRT1 in these native substrates and consequently provided evidence that resveratrol might activate SIRT1 indirectly [40]. This theory is supported by El-Mowafi et Alkhalaf, who gave a first hint that the effects of resveratrol may be mediated by cyclic adenosine monophosphate (cAMP) [42]. Performing in vivo and in vitro experiments, Park et al. discovered, that resveratrol might actually be a competitive inhibitor of cAMP-degrading phosphodiesterase (PDE) [43]. In the presence of low dose (≤50 μM) resveratrol, intracellular levels of cAMP in C2C12 myotube cells were significantly elevated. The elevation of cAMP levels further resulted in the activation of the Ca(2+)/calmodulin-dependent protein kinase kinase-β-AMP-activated protein kinase (CamKKβ-AMPK) pathway. AMPK then led to an increase of NAD+ levels, followed by SIRT1 activation and deacetylation of SIRT1 target proteins. These findings illustrate that cAMP might actually mediate the numerous effects of resveratrol and SIRT1 is hence activated indirectly [43]. To understand how resveratrol inhibits PDEs, the authors measured the effect of cAMP and resveratrol on the kinetics of recombinant PDE3 activity. At high concentrations of cAMP, there was no more inhibitory effect of resveratrol on PDE3. The incubation of PDE3 with the fluorescent cAMP analog 8-azido[DY547]-cAMP, which cross-links to its binding site when stimulated with UV, in the presence of increasing concentrations of either resveratrol or cAMP, confirmed that resveratrol is a competitive inhibitor of PDEs. To confirm that resveratrol increases cAMP levels also in vivo, the authors administered resveratrol to mice and measured cAMP levels in skeletal muscles and white adipose tissue. An increase of cAMP levels was indeed found in both tissues [43].

**Figure 1:**
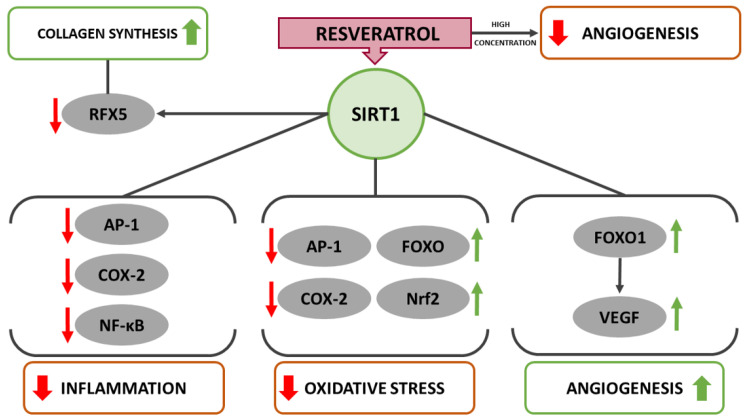
Schematic diagram of the potential role of resveratrol-induced SIRT1 activation in suppressing inflammation as well as oxidative stress and promoting angiogenesis in wound healing.

Once SIRT1 is activated it deacetylates several transcription factors that contribute to cellular regulation, such as forkhead box O 3 (FOXO3), nuclear factor kappa-light-chain-enhancer of activated B-cells (NF-κB) and p53. The SIRT1-regulated pathways affect cell survival, metabolism, stress resistance, endothelial function, and circadian rhythm [33]. In the context of wound healing, SIRT1 was shown to exert anti-inflammatory, and anti-oxidant properties, to induce collagen synthesis and to ignite autophagy as well as angiogenesis [7,44,45,46,47]. The specific resveratrol induced pathways that are to a large extent allegedly mediated by SIRT1 are discussed in the following sections and illustrated in Figure 1.

## 4. Resveratrol Alleviates Inflammation through the NF-κB and MAPK Pathway

### 4.1. NF-κB-Pathway

Several authors have suggested that resveratrol’s therapeutical value for wound healing is primarily attributed to its anti-inflammatory effects [6]. Its anti-inflammatory potential might predominantly be based on inhibiting the pathway activity of the transcription factor NF-κB [48,49,50,51,52]. This inhibition may be accomplished via a resveratrol-mediated, probably indirect, activation of SIRT1 or/and a target further downstream [34,53,54]. NF-κB plays a significant role in inflammation and is responsible for the orchestration and coordination of the inflammatory response [55]. The NF-κB family includes five members—p105/p50 (NF-κB1), p100/p52 (NF-κB2), p65 (RelA), RelB, and c-Rel. Together they mediate the transcription of target genes by binding to a specific DNA element [56,57,58]. All members of the NF-κB family build homo or hetero dimers composed of of p65 and p50 or p52 subunits, which are bound to its endogenous inhibitor nuclear factor kappa B (IκB) [57]. Recently, two separate pathways have been found that activate NF-κB—the classic canonical and an alternative pathway [57,59]. The anti-inflammatory effects of resveratrol are based on the classical pathway by inhibiting IκB kinase (IKK) [49,50,51]. The signal transmission of the classical pathway is mediated by Toll-like receptors (TLRs), interleukin-1 receptor (IL-1R), tumor necrosis factor receptor (TNFR) and antigen receptors. TNF-α, lipopolysaccharides (LPS) and IL-1β serve as signaling molecules [56]. These stimuli lead to an activation of TGF-β-activated kinase 1 (TAK1) which induces the activation of the IKK complex. This activation leads to the phosphorylation of IκB—the inhibitor of NF-κB. The ubiquitination of IκB proteins results in the release and consequent nuclear translocation of NF-κB, where it binds to κB binding sites in the promoter regions of genes encoding pro-inflammatory inducible enzymes [57,59]. Once activated, NF-κB can induce the transcription of various pro-inflammatory cytokines, such as IL-1, IL-6, IL-12 and TNF-α, chemokines and inflammatory mediators in different types of innate immune cells [56]. Resveratrol seems to be able to inhibit the classic NF-κB pathway activity through downregulation of p65 and IκB phosphorylation [49,50]. The underlying mechanism is based on resveratrol’s inhibitory properties, which hinder the translocation of NF-κB-p65 subunits from the cytosol to the nucleus through the suppression of IκB phosphorylation and decomposition [6,51]. This resveratrol-mediated inhibition of the NF-κB activity can be induced in a dose-dependent manner by diverse triggers such as TNF-α [60,61], IL-1ß [62,63] and/or LPS [64]. Immunocytochemical analysis of the localization of the p65 subunit of the NF-κB complex showed a LPS stimulated decrease of cytosolic p65 levels. In resveratrol treated cells however, this decrease was blocked [52]. Additionally, resveratrol seems to downregulate the expression of TGF-β- 1, that is involved in many cellular processes including cell growth, cell differentiation, cell migration, apoptosis and cellular homeostasis and plays a major role in wound healing [65,66]. TGF-β1 promotes degradation of Iκ-B, which in turn leads to the activation of Activator Protein 1 (AP-1), and NF-κB [65]. Besides interleukins, high-mobility group box 1 (HMGB1) is another significant pro-inflammatory mediator and extracellular damage-associated molecular-pattern protein, of which the release and induction are NF-κB-depended [6,67]. Ma et al. demonstrated a resveratrol-mediated inhibition of LPS-induced translocation of HMGB1 from the nucleus to the cytoplasm and the translocation of NF-κB p65 from the cytosol to the nucleus and IκBα phosphorylation [6].

As resveratrol is an agonist of SIRT1, Yeung et al. investigated, whether SIRT1 is involved in the regulation of the transcriptional activity of NF-κB [53]. In an acetylation assay, the authors showed that SIRT1 interacts with the RelA/p65 subunit of NF-κB and inhibits its transcription by deacetylation of RelA/p65 at lysine 310 [53]. The priorly required resveratrol-SIRT1 activation occurs probably indirectly through the induction of cAMP signaling via Epac1, resulting in the activation of the CamKKb-AMPK pathway, as suggested by Park et al. [43]. Zhu et al. showed that in fibroblasts the activation of SIRT1 leads to an inhibition of TNF-α-induced inflammation [45]. The inhibition of NF-κB activation by resveratrol is not cell type specific. Hence, these findings are valid for various cell types [60] and are probably also relevant for wound healing.

### 4.2. MAPK-Pathway

Zhang et al. stated that in a LPS-induced mouse mastitis model resveratrolinduced inhibition of pro-inflammatory cytokines is not only regulated via the NF-κB signaling pathway but also the via mitogen-activated protein kinase (MAPK) [48]. MAPKs belong to a family of highly conserved intracellular signaling molecules that are subdivided into three major pathways: ERKs, p38 kinases and c-Jun-NH2-terminal kinases (JNKs 1, 2, and 3) [68]. Both pathways, NF-κB and MAPK, were repeatedly shown to modulate the expression of TNF-α and IL-1β and hence reduce inflammation [69,70]. In an experimental setting, pretreatment of resveratrol significantly decreased the expression of p38 and extracellular signal-regulated kinases (ERK) phosphorylation compared to the group treated with LPS only [48]. Manna et al. observed JNK and the upstream mitogen-activated protein kinase (MEK) to be potential targets of resveratrol [60]. According to the authors, resveratrol displays additional effects more downstream in the MAPK pathway. They observed a TNF-induced AP-1 activation in untreated cells, while in resveratrol-treated cells, no AP-1 activation was found [60]. AP-1 represents a transcription factor family that includes Jun, Fos, Maf (musculoaponeurotic fibrosarcoma) and the activating transcription factor (ATF) proteins. C-Jun and c-Fos form the AP-1 early response transcription factor [71,72,73]. Resveratrol was reported to reduce the AP-1 DNA-binding and transcription activity by downregulating the expression of tumor necrosis factor-ß (TNF-ß)-induced p-c-Jun, c-Jun, and c-Fos [74,75,76]. As these kinases are required for the activation of AP-1, the target may either be them or/and an upstream factor, as proposed by Zhang et al. [48,60]. A SIRT1 knockdown unraveled SIRT1 to be potentially involved in AP-1 signaling, as AP-1 transcriptional activity was clearly reduced [77]. 

## 5. Resveratrol-Induced COX-2 Inhibition Minimizes Inflammation and Oxidative Stress

**Figure 2:**
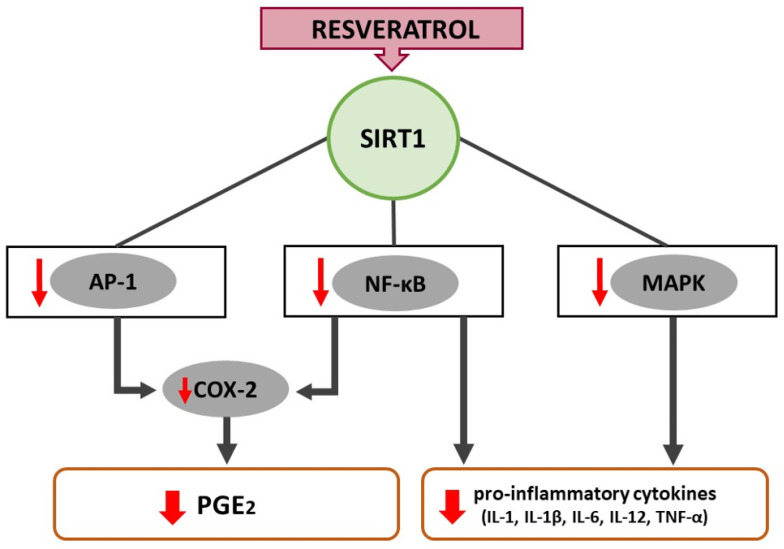
Resveratrol-induced signal transduction leading to an attenuated inflammatory response through downregulation of AP-1, NF-κB and MAPK.

Cyclooxygenase (COX) enzymes catalyze the conversion of arachidonic acid into prostaglandins (PGs). COX exists in 2 isoforms, known as COX-1 and COX-2 [78,79]. While COX-1 is expressed in most tissues and regulates homeostatic functions, COX-2 is expressed in response to physiological stimuli, including inflammation and oxidative stress [78,80]. There is evidence that COX-2 is also crucial for cell proliferation [81]. Resveratrol was shown to alleviate inflammation and oxidative stress by decreasing the levels of COX-2 [82]. The resveratrol-mediated suppression of COX-2 supports the theory of resveratrol as a useful treatment for inflammatory conditions [32]. The polyphenol is widely suggested as COX-2 inhibitor [83,84,85], while some authors claim resveratrol can inhibit both isoforms, COX-1 and COX-2 [86,87]. Resveratrol was reported to downregulate COX-2 at the transcriptional level and consequently suppress prostaglandin E (PGE) synthesis via modulation of the NF-κB, MAPKs, and AP-1 signaling cascades (Figure 2) [52,88,89]. Hence, the mechanism of action is comparable to resveratrol’s action on NF-κB– in the presence of resveratrol phosphorylation of IκB is suppressed and translocation of NF-κB-p65 subunits from the cytosol to the nucleus is consequently hindered [52]. Yang et al. found that resveratrol reduced the binding of the AP-1 early response transcription factors c-Jun and c-Fos to the COX-2 promoter. Moreover, the authors recognized that again, SIRT1-dependent mechanisms might be involved in the effects of resveratrol on COX-2. Rheumatoid arthritis synovial fibroblasts were incubated with resveratrol for 1 h and then challenged with bradykinin (BK). Resveratrol increased the expression of SIRT1 in a dose-dependent manner, leading to the down-regulation of the BK-induced COX-2 protein expression, mRNA levels, promoter activity, and PGE2 production [89]. However, whether SIRT1 contributes to resveratrol-mediated COX-2 modulation is still controversially discussed. While silencing of SIRT1 did indeed reverse the inhibitory potential of resveratrol against phorbol 12-myristate 13-acetate (PMA)-mediated NF-κB signaling pathway, it did not affect the inhibition of COX-2. These results suggest that the inhibitory potential of resveratrol on COX-2 is a SIRT1-independent event [90].

Jang et al. showed that not only inflammation but also oxidative stress can be reduced via the resveratrol-mediated inhibition of COX-2 [66]. The authors applied 2-O-tetradecanoylphorbol-13-acetate (TPA) to the skin of mice. TPA is known to induce oxidative stress, with significant accumulation of H2O2, enhanced levels of myeloperoxidase, oxidized glutathione reductase activities and decreases in glutathione levels and superoxide dismutase activity. As a response, TPA treatment elevated the expression of COX-1, COX-2, c-Myc, c-Fos, c-Jun, TGF-β1 and TNF-α. Topical treatment of mouse skin with resveratrol led to a decrease of H2O2, glutathione, myeloperoxidase, oxidized glutathione reductase and superoxide dismutase activities levels towards the solely TPA treated controls. At the highest dose tested, resveratrol dramatically reduced c-Fos expression and completely suppressed TGF-β1 expression. Resveratrol itself did not alter the expression of any other investigated genes. The anti-oxidative effects might possibly be induced by modulating the expression of c-Fos and TGF-β1 [66].

## 6. Resveratrol Interferes with Redox Balance through Upregulation of FOXO and Nrf2

**Figure 3:**
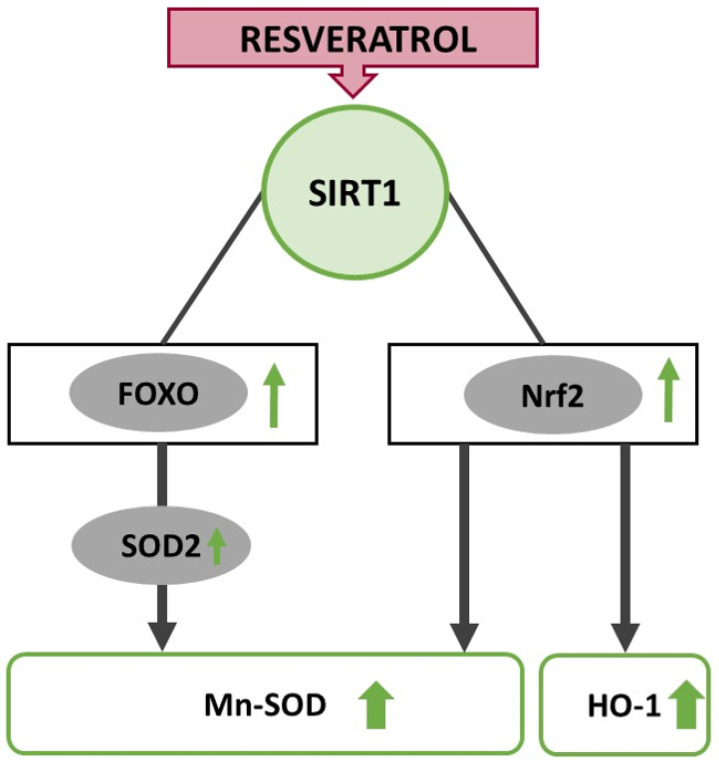
Resveratrol-induced signal transduction leading to reduced oxidative stress levels through upregulation of FOXO (FOXO1, FOXO3a and FOXO4) and Nrf2.

Oxidative stress results from an imbalance in the generation of reactive oxygen species (ROS) and the anti-oxidant production in cells (Figure 3). Enhanced oxidative stress damages macromolecules and impairs their functions, inducing inflammation, dysregulation of mitochondrial function, and cell death [91]. Therefore, excessive ROS production plays a crucial role in the orchestration of wound healing [92]. Resveratrol has been highlighted as a powerful anti-oxidant that inhibits ROS overproduction and protects the cells against hydrogen peroxide-induced (H_2_O_2_) oxidative stress [93,94]. Orihuela-Campos et al. showed in an H_2_O_2_-induced oxidative stress model on gingival fibroblasts that a concentration of 50 µM of resveratrol inhibited ROS production and led to an increase in cell proliferation and stimulation of mitochondrial respiration [95]. The anti-oxidative properties of resveratrol seem to be dosage-dependent; low to medial doses are associated with a reduction of oxidative stress levels and stabilized cell proliferation, whereas high concentrations may induce an increase in ROS levels [96]. Moreover, resveratrol shows better anti-oxidative activity in an environment with high levels of oxidative stress. In an in vitro model, the sole application of resveratrol did not alter oxidative stress levels, but induced cell proliferation. Only the combination of resveratrol and H_2_O_2_ exhibited protective effects against oxidative stress. Nevertheless, Kaleci and Koyuturk et al. showed that resveratrol could not fully eliminate H_2_O_2_ effects and did not reach levels of the control group in vitro [97]. The anti-oxidative properties of resveratrol are partially attributed to the direct effects of resveratrol as a radical-scavenger, but also indirect effects were observed, as resveratrol is able to modulate cellular anti-oxidant pathways [98]. 

### 6.1. Nrf2-Pathway

SIRT1 enhances the expression of nuclear factor erythroid 2–related factor 2 (Nrf2), which plays an essential role in maintaining the redox balance. The expression of Nrf2 has been observed throughout various human tissues and is also involved in skin homeostasis. Nrf2 is discussed as a crucial transcription factor that affects the anti-oxidative stress defense system. The activation of Nrf2 mediates anti-oxidant enzymes, contributing to epidermal cell protection from oxidative damage [99]. The role of the Nrf2 signaling pathway in resveratrol-mediated skin protection has been reported in several in vitro studies [100], but also showed promising results in vivo. So far, most studies investigating the anti-oxidative effects of resveratrol were conducted in the context of photo-aging; since skin integrity plays a crucial role in both, wound healing as well as photo-aging, the results of these studies might be equally relevant in the context of wound healing. In mice, the oral administration of 2 mg resveratrol per kg body weight attenuated UVB-induced epidermal thickening and provided effective protection from wrinkle formation induced by UVB irritation treatment. These protective effects regarding photo-aging are ascribed to the activation of the Nrf2 signaling pathway. In the resveratrol-treated group, Nrf2 was first suppressed by UVB irradiation but remained more resistant and was maintained at a higher level along with heme-oxygenase-1 (HO-1) compared to controls. Nrf2-dependent anti-oxidant enzymes such as HO-1 were induced by resveratrol in the liver and skin and inhibited MMPs. Therefore, the authors assume that resveratrol attenuates UVB-induced photo-aging by activating the Nrf2/HO-1 signaling pathway [101]. Zhou et al. investigated the anti-oxidative effects of resveratrol in wound healing. They found that resveratrol upregulated of Nrf2 and Mn-SOD expression inhuman umbilical vein endothelial cells. Nrf2 and Mn-SOD expression was as well upregulated in vivo in a diabetic rat model with burn injuries after resveratrol treatment. Since Mn-SOD belongs to the most important members of the antioxidant defense system that detoxifies superoxide anions under Nrf2 regulation, the authors assume that the anti-oxidative effects of resveratrol in wound healing occur through the promotion of Nrf2 aggregation, stimulating the expression of Mn-SOD as a downstream product [102]. Another described mode of action is the binding of Nrf2 to Kelch-like ECH-associated protein-1 (Keap1) that promotes the degradation of Nrf2 through the ubiquitin-proteasome pathway. In case of oxidative stress, Keap1 phosphorylates and activates Nrf2, which is then translocated to the nucleus and interacts with anti-oxidant response elements (ARE), promoting the expression of cytoprotective target genes, namely glutathione-S-transferases (GST), NAD(P)H: quinone oxidoreductase (NQO1), NQO2, γ-glutamylcysteine synthase (γ-GCS), glucuronosyltransferase and ferritin [103].

### 6.2. FOXO-Transcription Factor Family

The forkhead box O (FOXOs) transcription factor family includes FOXO1, FOXO3a, and FOXO4 and regulates apoptosis upon cellular stress, but are equally important for cell survival [104]. They activate ROS-detoxifying enzymes such as superoxide dismutase 2 (SOD2/Mn-SOD) and catalase. There is only vague evidence that the effect of SIRT1 is linked to the FOXO function; nevertheless, authors assume that SIRT1 regulates FOXO transcription factors by deacetylation. SIRT1 might enhance FOXO’s activity by nuclear translocation and regulating the gene-specific transcription. Brunet et al. declared that the translocation of FOXO3a from the cytoplasm to the nucleus is triggerd through deacetylation induced by SIRT1 in response to oxidative stress [105]. SIRT1 simultaneously promotes the expression of FOXO target genes involved in stress resistance, and decreases the transcription of genes involved in apoptosis. Thus, SIRT1 shifts the FOXOs-dependent response from apoptosis towards cell survival and stress resistance [105]. These results are in line with the study by Yun et al., conducted in human monocytic cells exposed to mannitol to mimic a diabetic milieu for elevated oxidative stress levels [106]. The hyperglycemic conditions lead to a significant decrease of SIRT1 and FOXO3a expression and elevated ROS production compared to normoglycemic cells; resveratrol treatment reversed this effect by inducing SIRT1 and subsequently FOXO3a expression, concomitant with reduced superoxide production [106]. In a p53-independent cell protective pathway Hori et al. found, that FOXO1, FOXO3a, and FOXO4 are involved in a resveratrol-induced upregulation of SOD2 [104]. The authors showed that a knockdown of these three FOXOs impeded SOD2 induction, ROS reduction, and anti-apoptotic function of resveratrol. These results indicate that all three FOXOs are indispensable for SIRT1-dependent cell survival and protection against oxidative stress [104]. Nevertheless, concrete prove whether FOXO upregulation is truly achieved by SIRT1 deacetylation is still elusive and further research is needed. 

## 7. Resveratrol Ignites Autophagy

Autophagy is a “self-digestive” process that protects cells against stressful conditions and allows the cells to adapt to environmental and/or developmental changes [107]. Autophagy not only prevents the accumulation of damaged cells but may also be crucial for regulating the inflammatory response and thus wound healing [108,109]. Lei Qiang et al. showed that wound healing in mice significantly relies on keratinocyte autophagy. Deficiency of epidermis-specific autophagy in mice inhibited wound closure, re-epithelialization, keratinocyte proliferation and differentiation, dermal granulation tissue formation, and infiltration of immune cells including macrophages, neutrophils, and mast cells [110]. The autophagy-inducing effect of resveratrol was confirmed by Josifovska et al., who observed lower cell death rates, increased LC3II/I ratio and decreased p62 expression in ARPE-19 cells treated with resveratrol [111]. Even though there is a paucity of information about the autophagy-inducing effects of resveratrol in cutaneous wounds, it appears plausible that the same mechanisms support wound healing. There is evidence that SIRT1, as a vital regulator of cell survival, may be involved in resveratrol-mediated autophagy [112]. SIRT1-induced autophagy was shown to play a key role for the protective effects of CR and resveratrol supplementation in the liver of rats [113]. In osteoporotic rats, high-dose-resveratrol administration promoted an increased SIRT1-expression in osteoblasts compared to the controls. Autophagy was assessed by the formation of autophagic vacuoles in transmission electron microscopy. There were more mitophagome vacuoles in the resveratrol group than in the control group. Additionally, Akt phosphorylation and mTOR phosphorylation were down-regulated in the resveratrol-treated groups. The phosphorylation of Akt has been suggested as one of the indicators of PI3K/Akt pathway activation. The results provide evidence that PI3K/Akt signaling pathway is involved in resveratrol-mediated autophagy. According to this study, resveratrol treatment displayed no effects on p-p38 and p-JNK activities in osteoblasts [112]. In contrast to these results, other authors found that resveratrol induced autophagy not only via inhibiting Akt/mTOR, but also via activating the p38-MAPK pathway. However, the exact mechanisms are not yet elucidated [114,115].

## 8. Resveratrol May Increase Collagen Synthesis through Deacetylation of RFX5

While the anti-oxidative and anti-inflammatory potential of resveratrol is widely accepted, there is a debate concerning the effects of resveratrol in cutaneous repair processes such as collagen deposition, granulation tissue formation, and tissue remodeling. Hunt et al. stated that calorie-restricted rats healed at a slower pace than ad libitum fed rats. The authors argued that adequate nutrition is inevitable for the complex processes involved in wound healing [116]. In contrast to these results, Reed et al. showed that over long term, CR promoted the synthesis of structural proteins and trophic factors required for wound repair [117]. The question rises, if the same accounts for the CR-mimetic resveratrol. In an in vivo experiment, Christovam et al. found that both, resveratrol and CR equally increased fibroblast maturation and collagen deposition in excisional lesions on the dorsum of rats [7]. An increase of type I collagen was observed on day 3 and 10 in the resveratrol-treated group. An enhanced number of blood vessels, VEGF, fibroblasts and birefringent collagen fibers were found in the areas of the lesion. The authors assumed that these effects may be ascribed to the action of SIRT1 [7]. At least in smooth muscle cells SIRT1 was shown to be involved in the regulation of collagen transcription by NAD-dependent deacetylation of the regulatory factor for X-box (RFX5) [118]. RFX5 binds to the collagen type I (COL1A2) gene’s transcription start site and represses its transcription. SIRT1 might be able to disrupt the repression of the COL1A2 promoter activity by RFX5 [118]. By doing so, SIRT1 might promote collagen transcription and ultimately aid in restoring tissue integrity in lesions.

## 9. Resveratrol Exerts Dual Effects on Angiogenesis

Angiogenesis plays a significant role in tissue repair. VEGF is a potent mediator of vascular endothelial cells and plays a crucial role in regulating physiological angiogenesis [119]. It has been shown that resveratrol has contrasting effects on angiogenesis; depending on several factors, resveratrol exerts either pro-angiogenetic or anti-angiogenetic effects. The dosage of resveratrol and the cell type is shown to influence its differential effects on angiogenesis [120]. Pro-angiogenic effects were detected in tissues affected by ischemia-reperfusion injury, such as a peri-infarct in the myocardium [121], while anti-angiogenic effects were observed during tumor growth [122].

### 9.1. Pro-Angiogenic Effects of Resveratrol

Perfusion-enhancing effects of resveratrol have been shown by several in vitro and in vivo studies [7,123,124]. It has been observed that its pro-angiogenic potential relies to a large extent on SIRT1-mediated deacetylation of FOXO1 [125,126]. FOXO1 then activates ROS-detoxifying enzymes and is hence involved in the oxidative stress response. As oxidative stress can disturb angiogenesis [127], resveratrol’s pro-angiogenic effects might partially be ascribed to its anti-oxidative potential. Huang et al. showed that resveratrol promotes diabetic wound healing via SIRT1-FOXO1-c-Myc signaling pathway mediated angiogenesis [47]. FOXO1 is an essential downstream molecule of SIRT1 and a crucial checkpoint of endothelial growth, which can limit vascular expansion [126]. FOXO1 directly regulates VEGFA expression, which is one of the most potent pro-angiogenetic factors in wound healing. In vitro and in vivo experiments showed that FOXO1 is vital for VEGF transcription and expression and subsequently to sprout new blood vessels [128]. c-Myc regulates the mitochondrial function of vascular endothelial cells and is negatively regulated by FOXO1 [129]. Hyperglycemia-induced disturbance of angiogenesis plays a crucial role in the pathogenesis of diabetic non-healing skin ulcers. In diabetic patients, hyperglycemia leads to a decrease of SIRT1 expression in endothelial cells and endothelial dysfunction, which subsequently impairs the angiogenic function of endothelial cells. Under hyperglycemic conditions, resveratrol modulates SIRT1 c-Myc expression by promoting FOXO1 degradation [47]. This results in endothelial protection due to an improvement of endothelial cell survival and function [47].

### 9.2. Anti-Angiogenic Effects of Resveratrol

Previous studies described the effects of resveratrol regarding angiogenesis as a double-edged sword, whereas the dosage seems to be the determining factor. Anti-angiogenic effects of resveratrol were mainly observed in tumors; the inhibition of angiogenesis is associated with anticancer effects, which moved the agent in the focus of extensive research. Bråkenhielm et al. demonstrated an inhibitory effect of resveratrol on angiogenesis by suppressing VEGF receptor-mediated capillary endothelial cell growth in vitro and in vivo. This anti-angiogenic effect diminished in a dose-dependent manner [130], which aligns with Wong and Fiscus’s findings: only high concentrations of resveratrol suppressed the cytoprotective pathway NO/cGMP/PKG-1. This pathway usually protects endothelial cells against spontaneous apoptosis; hence, the high concentrations of resveratrol inhibited endothelial cell proliferation and tube formation and subsequently angiogenesis, which might be related to the overall anticancer effects of resveratrol [122].

## 10. Clinical Value of Resveratrol

Inflammation, inadequate perfusion, oxidative stress and impaired wound healing are indivisibly linked [92,131]. Resveratrol has the potential to improve wound healing due to its anti-inflammatory, pro-angiogenic and anti-oxidative properties [24,36,47,132]. Oral administration of 500 to 1000 mg of resveratrol was shown to be able to minimize oxidative stress by reducing ROS and to increase total anti-oxidative capacity (TAC) in healthy patients, as well as in patients with diabetes [29,133]. Resveratrol appears to have a positive systemic influence on oxidative stress and pro-inflammatory markers, which in turn may positively influence wound healing [26,28,29,30,31,92]. In clinical trials, a daily dose up to 1000 mg resveratrol for 3 months seemed to exhibit anti-inflammatory and anti-oxidant properties without any adverse effects [134]. Topical application of a resveratrol-loaded dermal matrix in diabetic foot ulcers showed accelerated wound healing by inhibiting the inflammatory response, decreasing oxidative stress, and promoting angiogenesis. Despite its numerous beneficial effects in wound healing, the clinical use of resveratrol as a pharmaceutical drug still faces several limitations; its poor bioavailability and fast metabolism rate are considered as the most limiting [39]. Although, in vitro studies reported a high efficacy of beneficial effects of resveratrol, the distribution in tissues is significantly reduced [135]. For example, 25 mg intake of resveratrol resulted in plasma concentrations lower than 10 ng/mL; a high dosage of 5000 mg reached plasma concentrations of 500 ng/mL [136]. Resveratrol also shows low water solubility (<0.05 mg/mL), which impairs its absorption. It has been shown that both, stability and water solubility are strongly affected by pH and temperature; solubility at a pH of 1.2 is 64 µg/mL, whereat it changes to 61 µg/mL at a pH of 6.8 and 50 µg/mL above pH 7.4. Once solubilized in water, only under acidic conditions resveratrol is stable at room or body temperature. With increasing pH, the stability of resveratrol decreases by degrading the stilbene exponentially. Therefore, resveratrol seems most stable at a low pH and low temperatures, no exposure to solar radiation and oxygen, and in liquid form [137]. To overcome the low water solubility and bioavailability of resveratrol—which means lower need for high doses, as well as fewer adverse effects—more complex formulations and different kind of drug carriers were developed and evaluated for clinical eligibility. Therefore, nano- and micro-encapsuled systems were designed to achieve improvements in skin delivery of resveratrol. For instance, solid lipid nanoparticles have been tried and tested as novel drug carriers that can incorporate lipophilic agents and improve its stability, bioavailability, water solubility, and biocompatibility [138]. In general, skin absorption of resveratrol depends on the vehicle in which it is formulated. Hung et al. reported a superior permeation of resveratrol with an aqueous solution compared to an oily system [139]. To improve resveratrol’s chemical stability in cosmetic formulations, lipophilic derivatives such as resveratryl triacetate are designed [140]. Results of recent studies highlighted that resveratrol-loaded dermal matrix systems seem to be promising wound dressings, as they release resveratrol directly at the target site. Especially the combination of specific wound dressing properties and resveratrol lead to synergistic beneficial effects in wound healing [141,142,143]. 

Regarding resveratrol’s safety, it seems to be a well-tolerated natural compound for both, oral intake, as well as topical application [144]. An acceptable daily intake of resveratrol was defined as 450 mg/kg [145]. Very high doses of resveratrol were associated with some adverse effects such as diarrhea, nausea, anemia, and abdominal discomfort [146]. Nevertheless, resveratrol can be considered as a safe, natural compound. Although there is little clinical evidence for the efficacy and applicability of resveratrol treatment in wound healing, the low number of clinical studies show promising results that resveratrol might be a feasible approach to support healing in dermal wounds. Resveratrol could consequently become a useful tool in modern wound therapy as an adjuvant to conventional wound therapy.

## 11. Conclusions

Resveratrol allegedly provides a broad range of beneficial effects for our skin. So far, the most promising benefits were reported for photo-aging and wound healing. Its benefits for cutaneous wounds seem to be numerous, whereas the scientific evidence is often scarce. The effect that is best supported is doubtlessly its anti-inflammatory potential via the modulation of NF-κB and MAPK pathways. However, the pathways for the described effects are partly entangled, which makes its understanding blurry and, therefore, challenging to draw defined conclusions [147]. According to the current state of science, SIRT1 seems to take a key role in several cascades of effects that prevent inflammation and oxidative stress and lead to improved wound bed perfusion and collagen transcription. Nevertheless, most studies fail to detect a direct target of resveratrol in the context of SIRT1 modulation. Park et al. finally achieved to unravel the mechanism of action and proposed that resveratrol might be a competitive inhibitor of PDEs [43]. On the other hand, a broad selectivity assessment against over 100 targets including receptors, enzymes, ion channels, and transporters revealed that resveratrol might be highly promiscuous [40]. As resveratrol seems to bind to a great range of macromolecular targets, the question arises, if it might be considered a frequent hitter. Then again, its wide range of action could also simply route in its anti-oxidative potential [127]. Not only due to its multivariate properties as a potential frequent hitter, but also because of its dosage-dependent effects, resveratrol appears as a two-sided sword; namely, low to moderate concentrations are associated with anti-oxidative properties, whereas higher concentrations elevate oxidative stress levels and induce cell death. Furthermore, the clinical use of resveratrol is limited by its poor bioavailability and fast metabolism rate. We believe that despite the outlined limitations, topically applied resveratrol may serve as a promising adjuvant agent to support wound healing, without any serious adverse effects. Further studies are still needed to determine its relevance for clinical applications, as well as to enhance our understanding of the exact molecular targets leading to resveratrol’s diverse benefit.

## Data Availability

Not applicable.
